# Supplementary figures and images for: Distinct cortico-striatal compartments drive competition between adaptive and automatized behavior
Source: PLoS One. 2023 Mar 21;18(3):e0279841. doi: 10.1371/journal.pone.0279841 (PMC10030038; doi:10.1371/journal.pone.0279841)

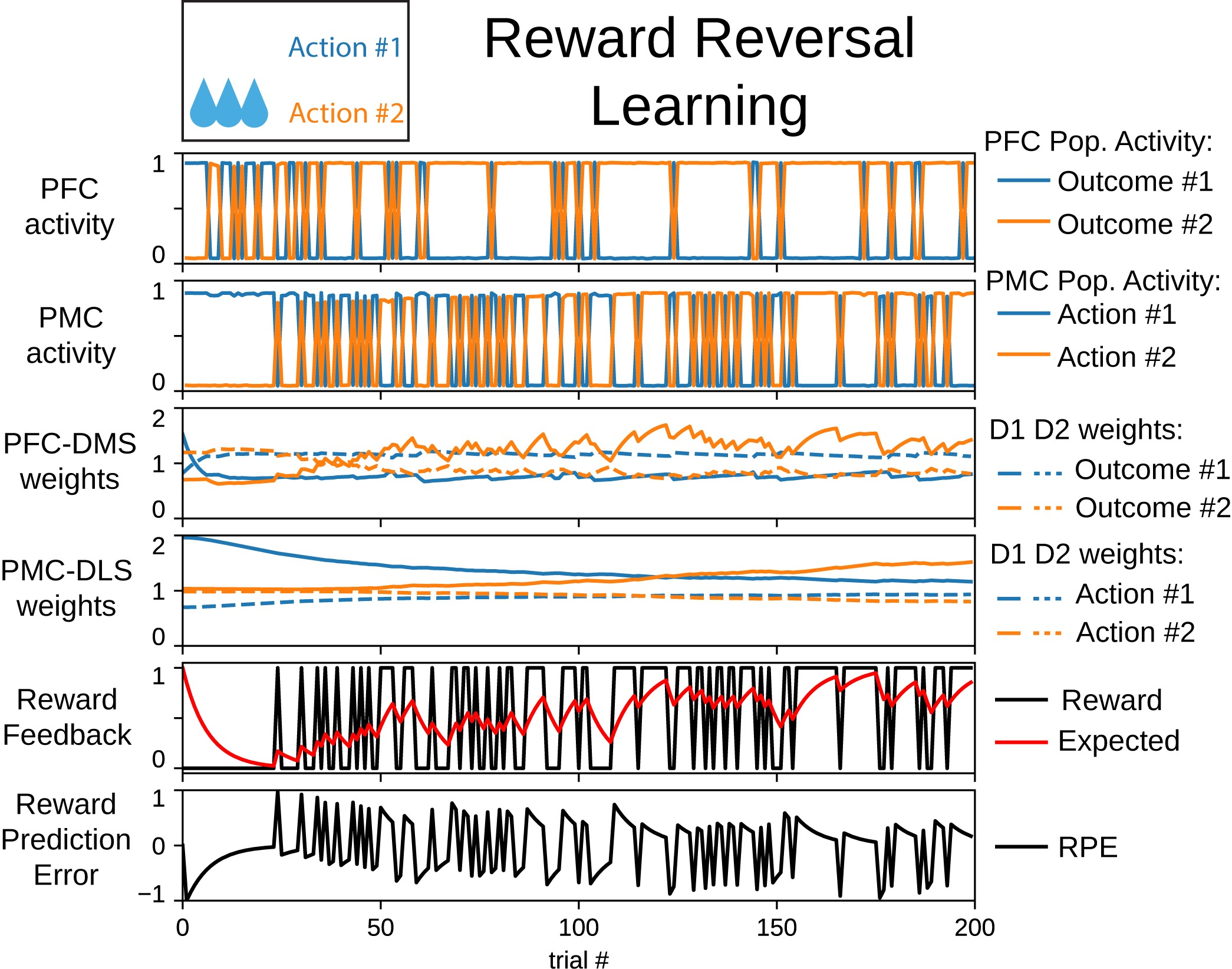

Supplement: S1 Fig — In panels depicting cortico-striatal weights, D1 synaptic weights are solid traces and D2 synaptic weights are dashed traces. Blue traces correspond to action #1 and orange traces correspond to action #2. (TIF) [file pone.0279841.s001.tif]

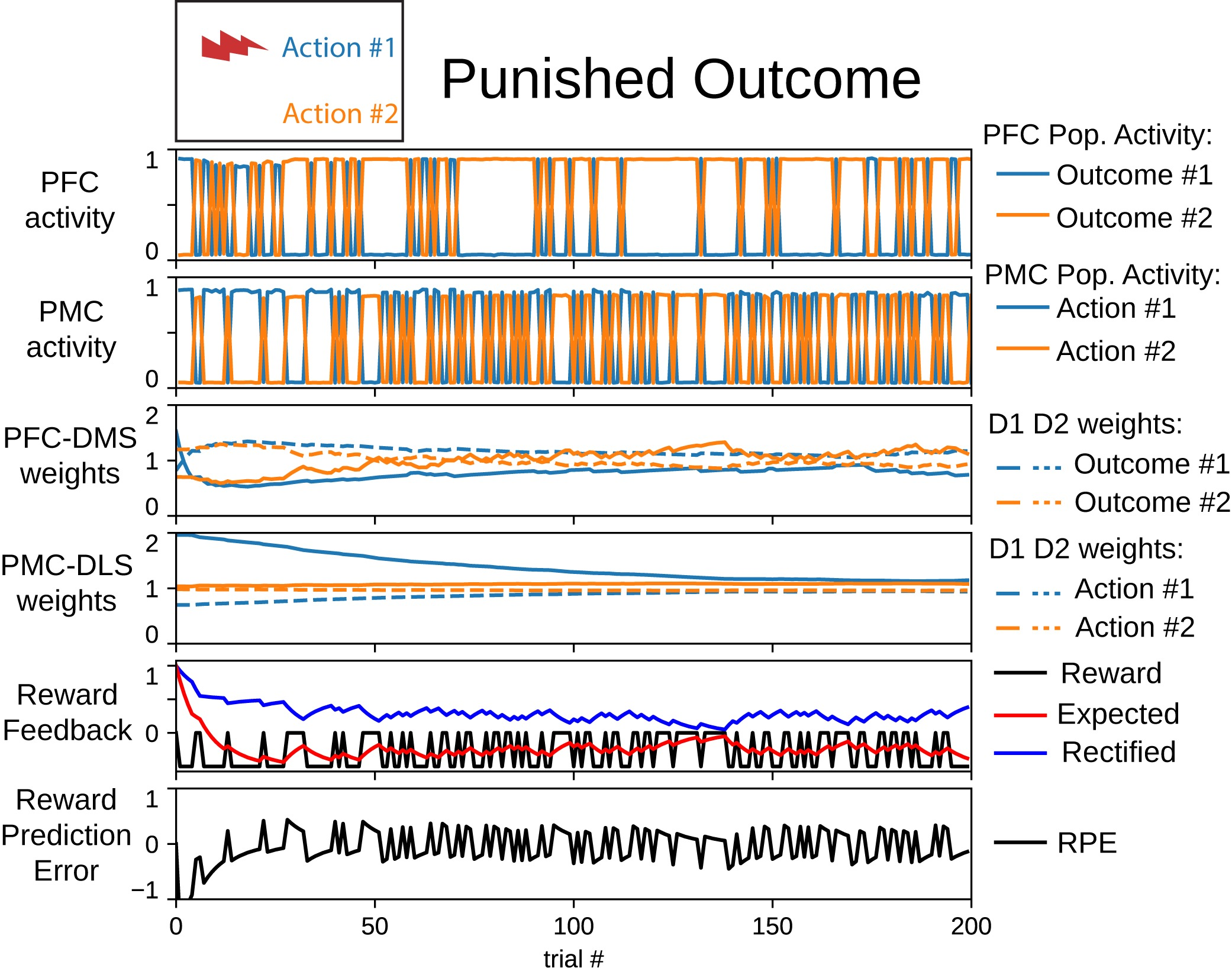

Supplement: S2 Fig — In panels depicting cortico-striatal weights, D1 synaptic weights are solid traces and D2 synaptic weights are dashed traces. Blue traces correspond to action #1 and orange traces correspond to action #2. (TIF) [file pone.0279841.s002.tif]

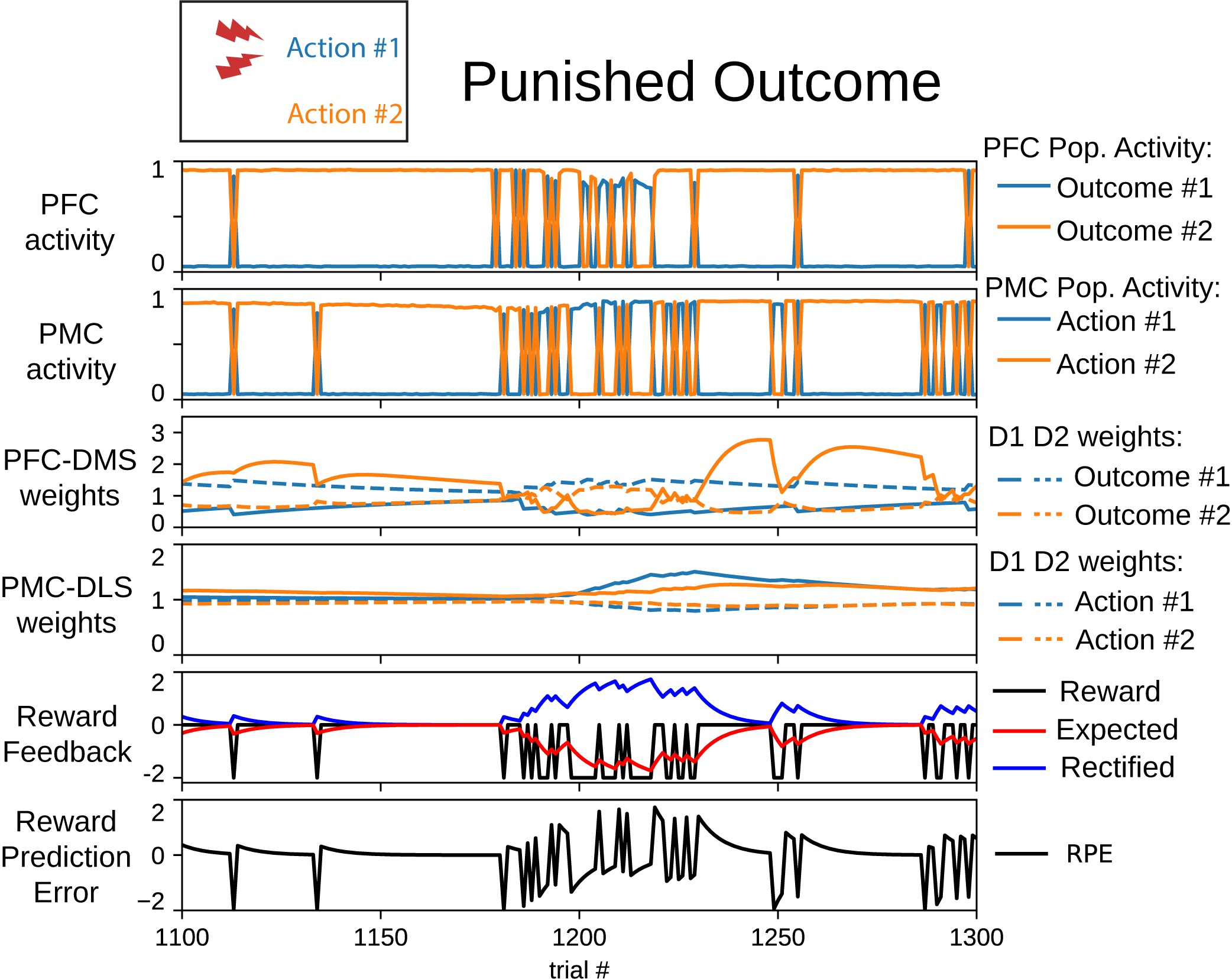

Supplement: S4 Fig — In panels depicting cortico-striatal weights, D1 synaptic weights are solid traces and D2 synaptic weights are dashed traces. Blue traces correspond to action #1 and orange traces correspond to action #2. (TIF) [file pone.0279841.s004.tif]
