# Supplementary material for: Distinct cortico-striatal compartments drive competition between adaptive and automatized behavior
Source: PLoS One. 2023 Mar 21;18(3):e0279841. doi: 10.1371/journal.pone.0279841 (PMC10030038; doi:10.1371/journal.pone.0279841)
Supplement: S3 Fig — Sessions with different values for the reward magnitude are shown here for the reward and punished outcome challenges, and the value of reward is indicated in each subfigure title. Among the reward and punishment, the values used for simulations in the main results are indicated by an asterisk (*). (PDF) [file pone.0279841.s003.pdf]

A. Initial Learning (R=1)

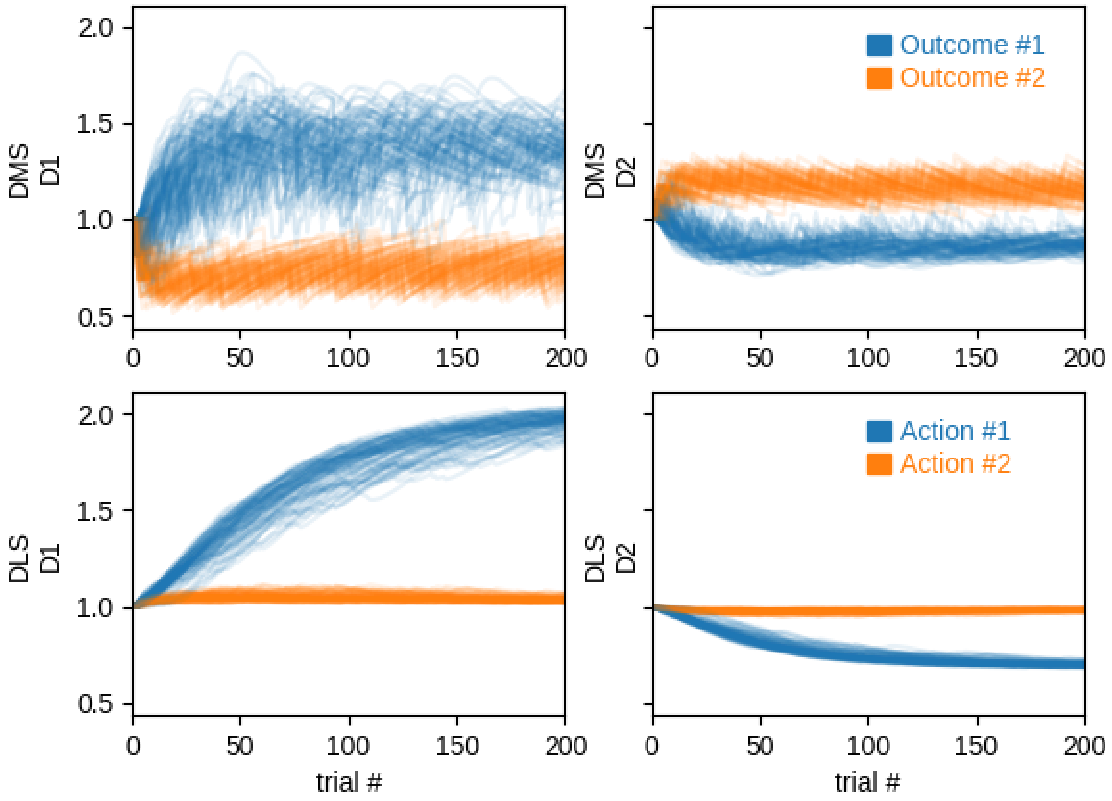

B. Devalutaion (R=0.2)

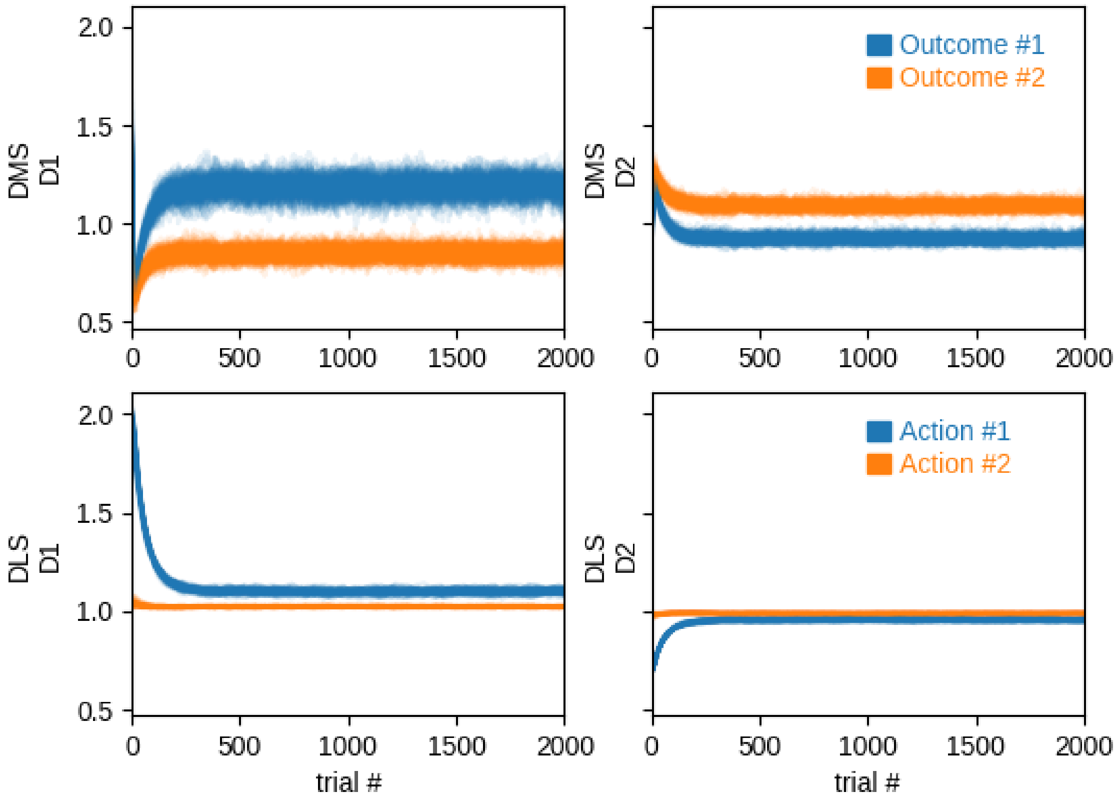

C. Reward reversal (R=0.2)

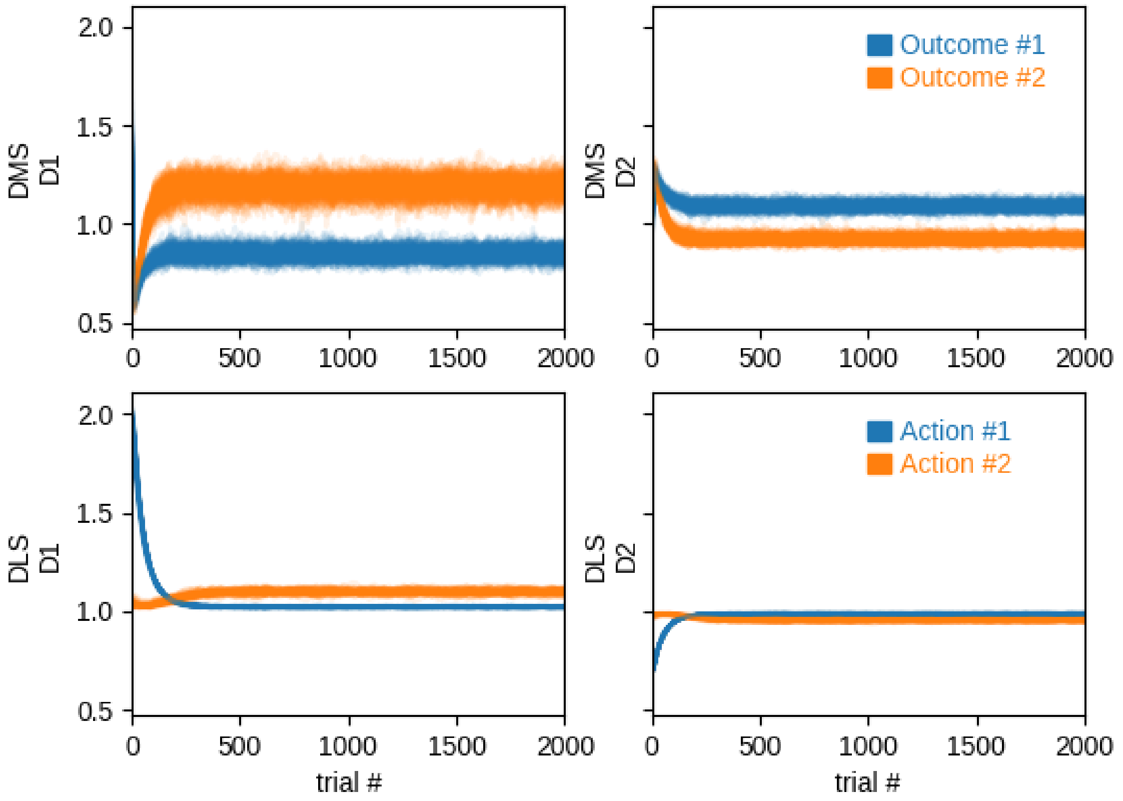

D. Reward reversal (R=0.5)

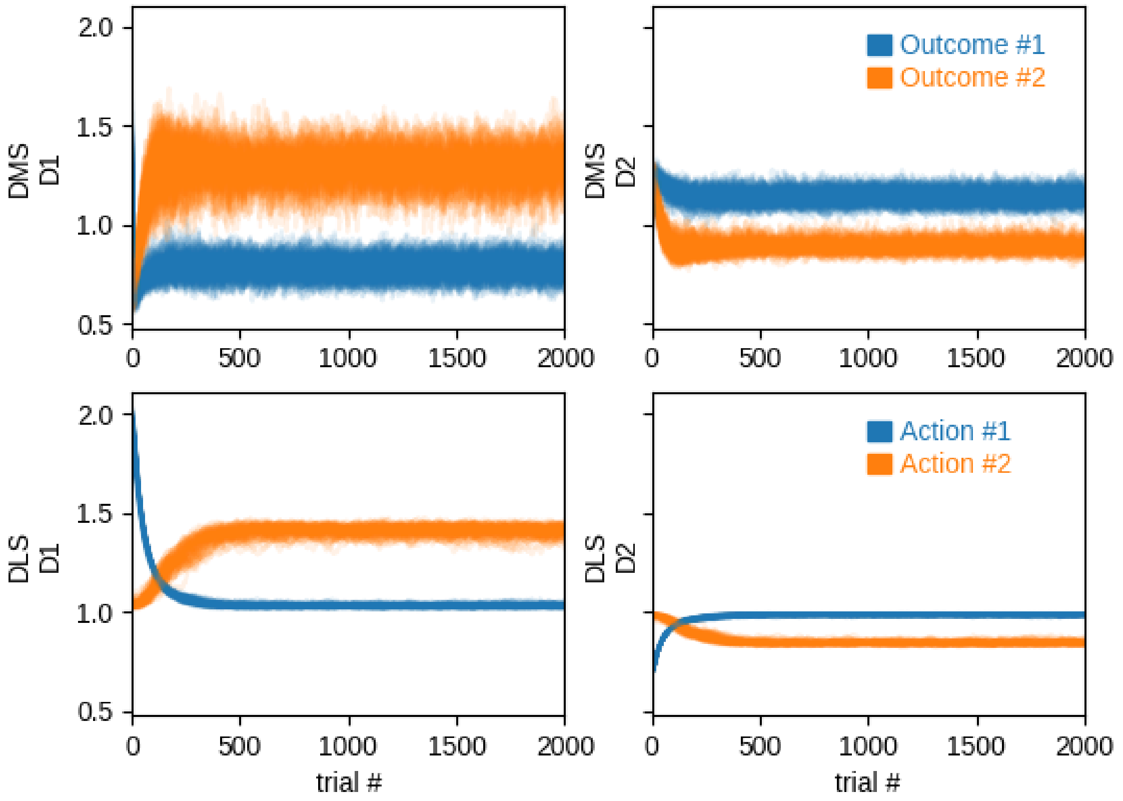

E. Reward reversal (R=1)*

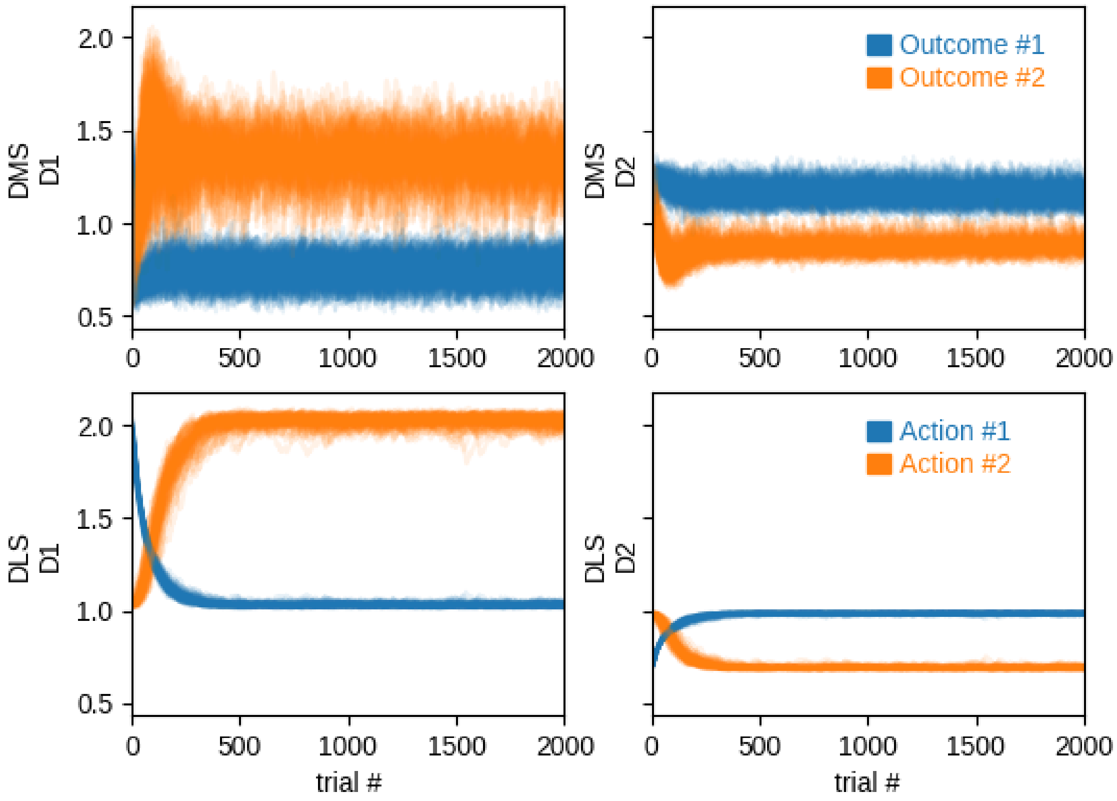

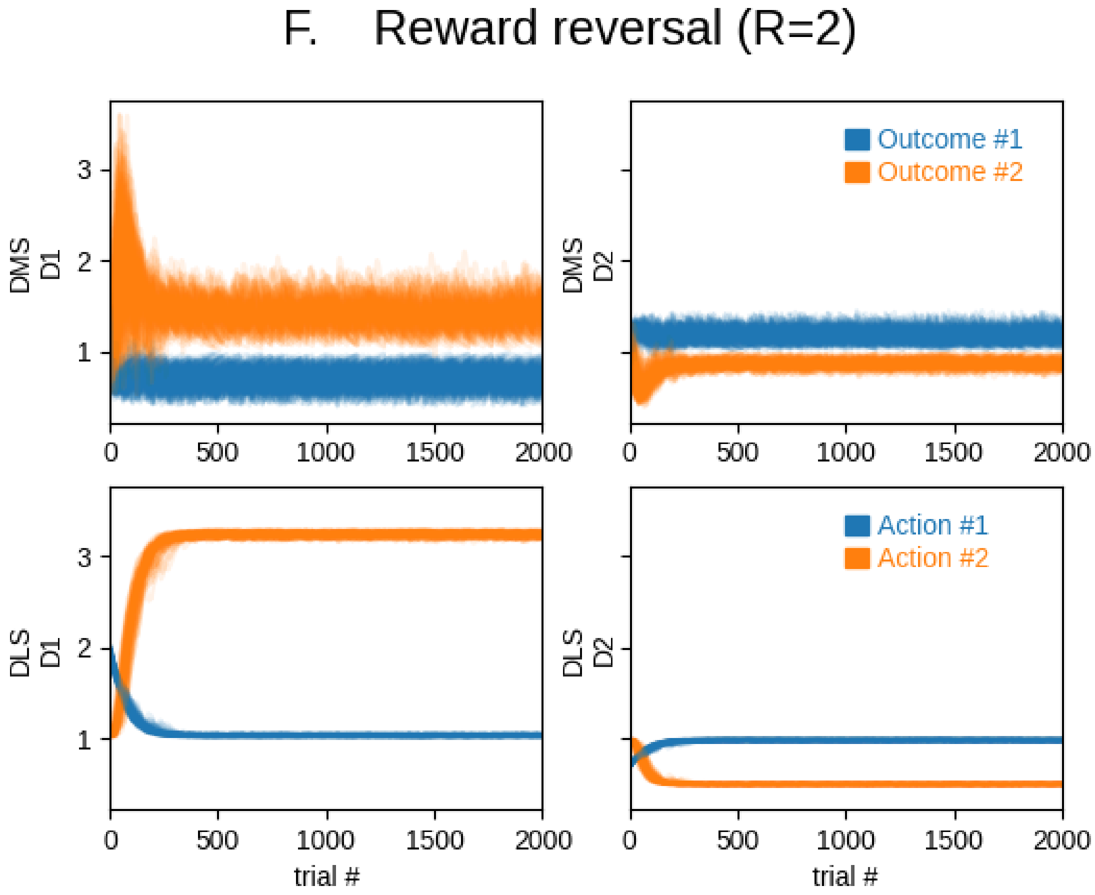
F. Reward reversal (R=2)
DMS D1
DMS D2
DLS D1
DLS D2
Outcome #1
Outcome #2
Action #1
Action #2
trial #
trial #

G. Punished outcome (R=-0.2)

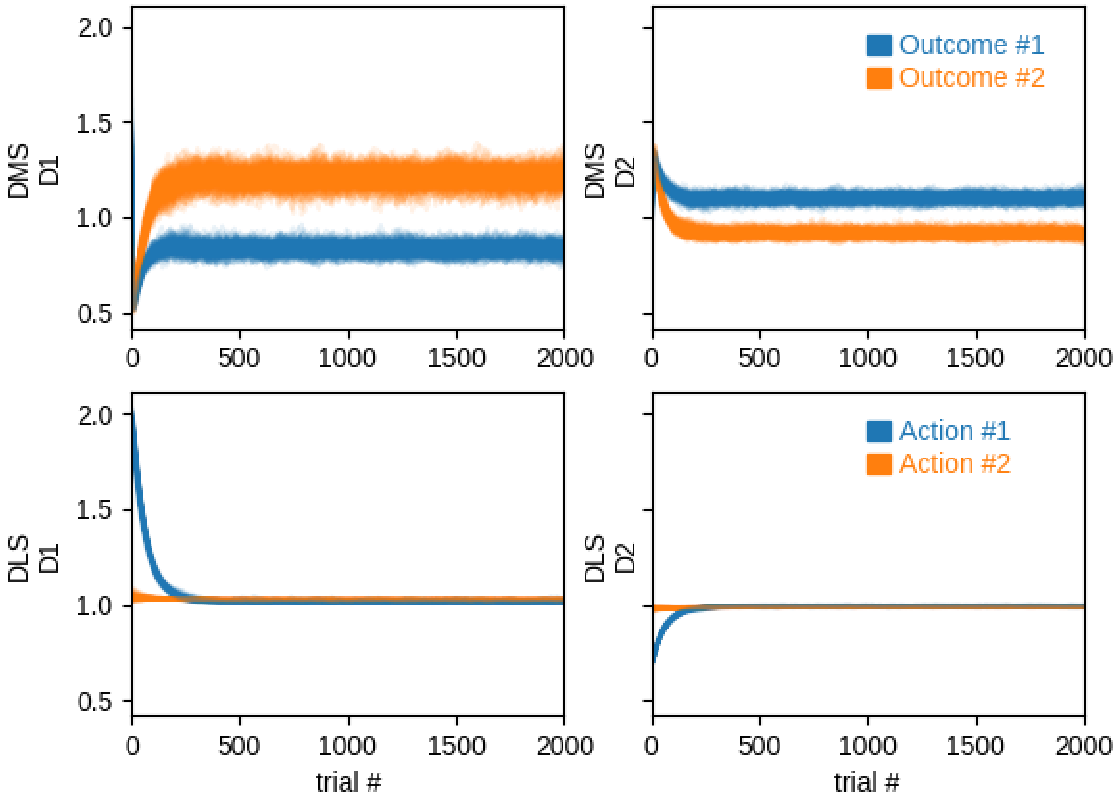

H. Punished outcome (R=-0.5)*

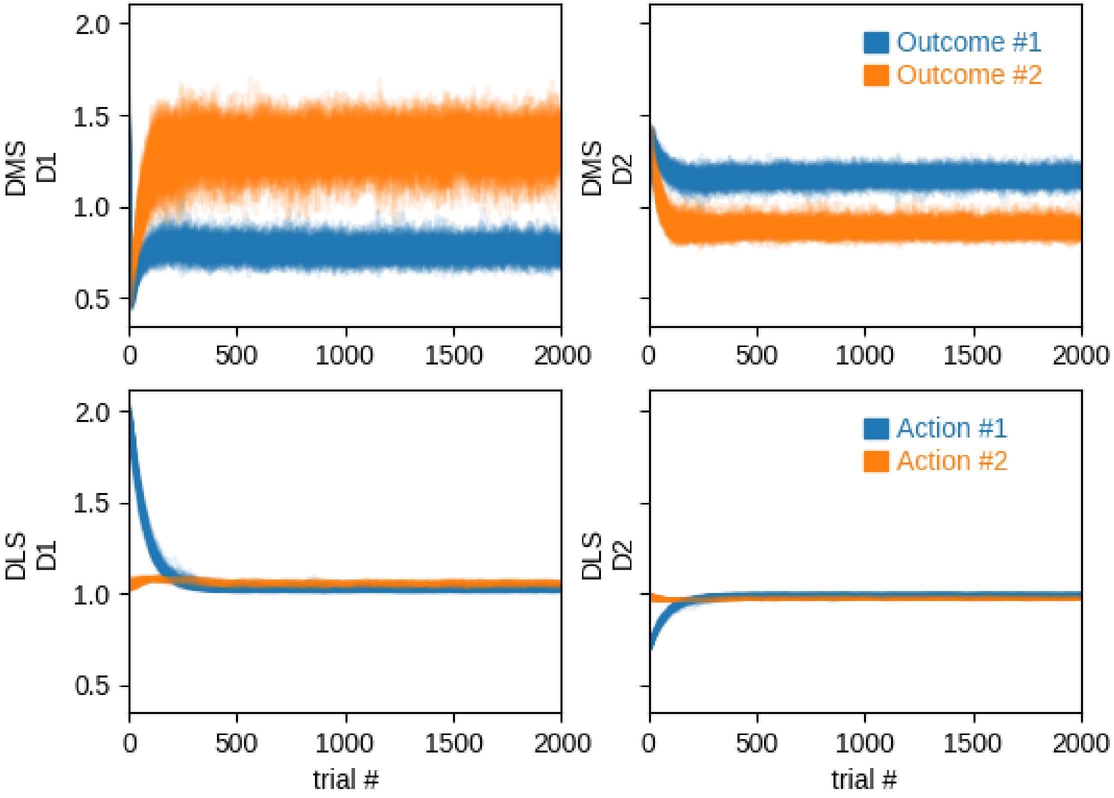

I. Punished outcome (R=-1)

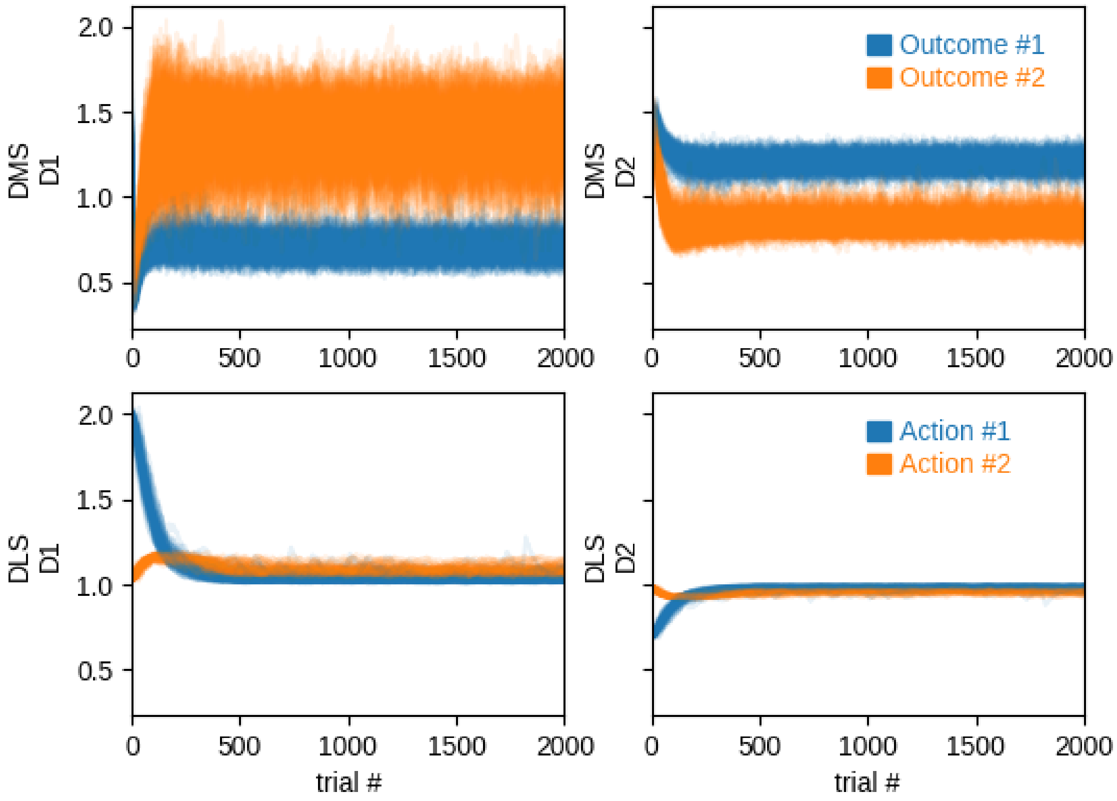

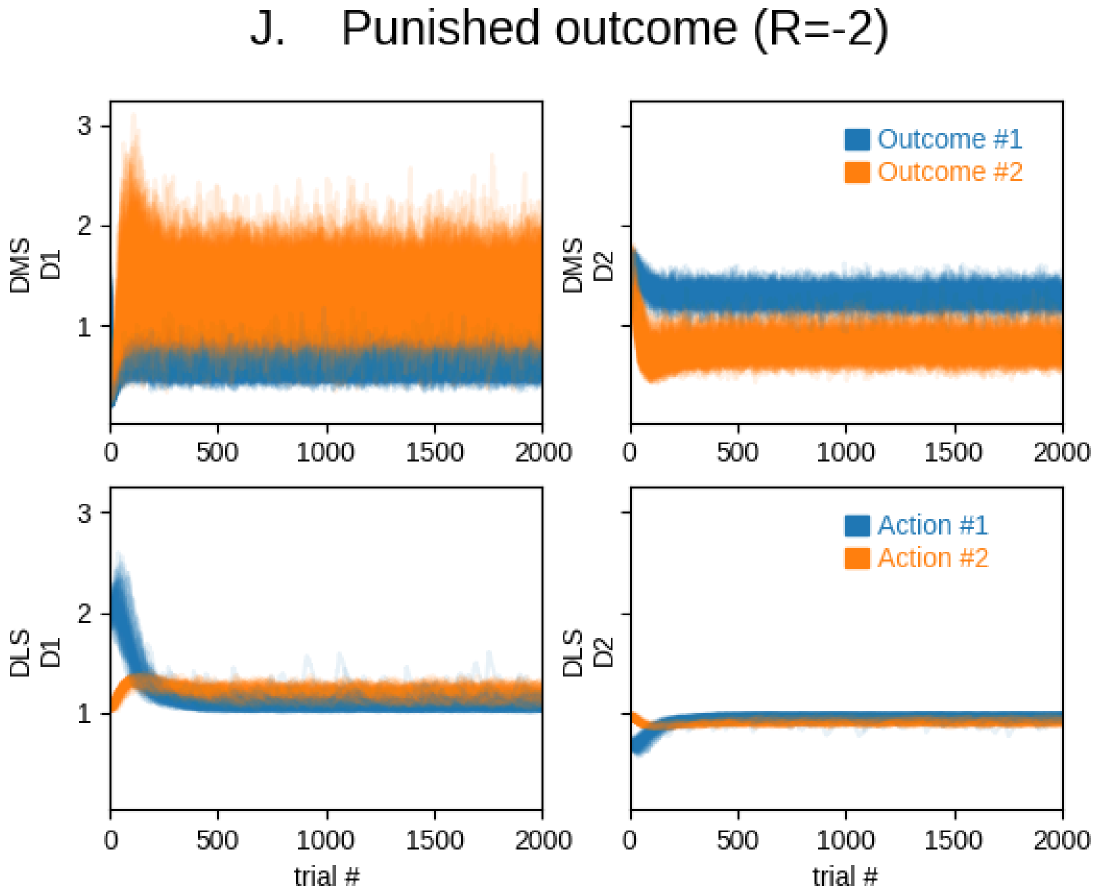
J. Punished outcome (R=-2)
DMS D1
DMS D2
DLS D1
DLS D2
Outcome #1
Outcome #2
Action #1
Action #2
0
1
2
3
500
1000
1500
2000
trial #
